# Reducing Emergency Department Transfers from Skilled Nursing Facilities Through an Emergency Physician Telemedicine Service

**DOI:** 10.5811/westjem.2020.7.46295

**Published:** 2020-10-08

**Authors:** Joshua W. Joseph, Maura Kennedy, Larry A. Nathanson, Liane Wardlow, Christopher Crowley, Amy Stuck

**Affiliations:** * Beth Israel Deaconess Medical Center, Department of Emergency Medicine, Boston, Massachusetts; † Massachusetts General Hospital, Department of Emergency Medicine, Boston, Massachusetts; ‡ West Health Institute, La Jolla, California

## Abstract

**Introduction:**

Transfers of skilled nursing facility (SNF) residents to emergency departments (ED) are linked to morbidity, mortality and significant cost, especially when transfers result in hospital admissions. This study investigated an alternative approach for emergency care delivery comprised of SNF-based telemedicine services provided by emergency physicians (EP). We compared this on-site emergency care option to traditional ED-based care, evaluating hospital admission rates following care by an EP.

**Methods:**

We conducted a retrospective, observational study of SNF residents who underwent emergency evaluation between January 1, 2017–January 1, 2018. The intervention group was comprised of residents at six urban SNFs in the Northeastern United States, who received an on-demand telemedicine service provided by an EP. The comparison group consisted of residents of SNFs that did not offer on-demand services and were transferred via ambulance to the ED. Using electronic health record data from both the telemedicine and ambulance transfers, our primary outcome was the odds ratio (OR) of a hospital admission. We also conducted a subanalysis examining the same OR for the three most common chronic disease-related presentations found among the telemedicine study population.

**Results:**

A total of 4,606 patients were evaluated in both the SNF-based intervention and ED-based comparison groups (n=2,311 for SNF based group and 2,295 controls). Patients who received the SNF-based acute care were less likely to be admitted to the hospital compared to patients who were transferred to the ED in our primary and subgroup analyses. Overall, only 27% of the intervention group was transported to the ED for additional care and presumed admission, whereas 71% of the comparison group was admitted (OR for admission = 0.15 [9% confidence interval, 0.13–0.17]).

**Conclusion:**

The use of an EP-staffed telemedicine service provided to SNF residents was associated with a significantly lower rate of hospital admissions compared to the usual ED-based care for a similarly aged population of SNF residents. Providing SNF-based care by EPs could decrease costs associated with hospital-based care and risks associated with hospitalization, including cognitive and functional decline, nosocomial infections, and falls.

## INTRODUCTION

Transfers from skilled nursing facilities (SNF) to the emergency department (ED) account for approximately 14 million ED visits annually, a fifth of which may be avoidable.^1^ In many cases, ED visits lead to admission, which in turn conveys risks of cognitive and functional decline, nosocomial infections, and falls.^2^,^3^ Furthermore, for the frailer subpopulation of SNF residents transferred to the ED, up to 78% of their resulting hospitalizations are potentially avoidable.^4^ Several solutions have been proposed to reduce admissions for these patients. One is to improve the quality of ED care for seniors and SNF residents through the development of geriatric-focused emergency care, and improved communication between SNFs and EDs.^5^ Incentive programs have also been established to improve longitudinal management of chronic medical conditions by SNFs, reducing transfers for patients with congestive heart failure (CHF) and diabetes mellitus (DM).^6^,^7^

Few studies have targeted the scenario that often triggers a transfer: when the SNF resident has an acute medical condition such as a fall, a fever, or an exacerbation of a chronic disease. Many SNFs retain on-call medical staff, but most lack the infrastructure to manage acute unscheduled care, particularly after-hours, and SNF healthcare teams often have little recourse other than to call 911 when patients need evaluation.^8^–^10^ One potential intervention to address this scenario is enlisting a physician via telemedicine to evaluate patients with acute care needs at the SNF. Telemedicine consults have been successfully used within EDs for a variety of subspecialties; providing rapid evaluations within the SNF setting could obviate transfers for minor injuries. Prompt evaluations could enable earlier interventions in acute infections and chronic disease exacerbations, potentially preventing the need for ED transfers or facilitating earlier transfers when warranted.

### Objectives

Our primary objective was to determine whether a SNF-based telemedicine consultation service staffed by emergency physicians (EP) could reduce hospital admissions of patients requiring acute evaluation, compared to patients who were taken directly to an ED. Our secondary objectives were to compare care escalation for conditions most amenable to on-site acute care in the SNF, and to broadly examine the financial implications of on-site acute care.

## METHODS

### Study Setting and Design

**Figure 1:**
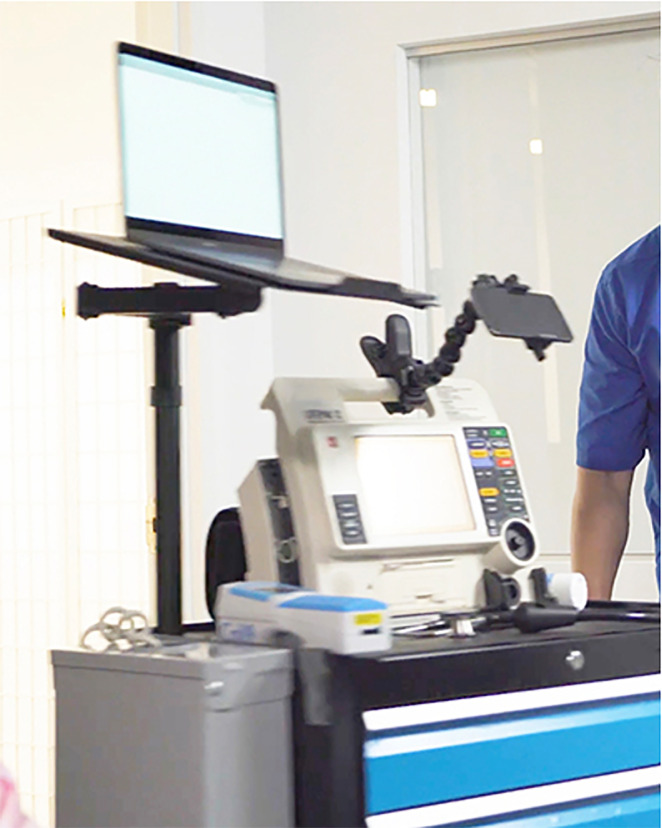
Clinical care specialist telemedicine cart in a skilled nursing care facility.

This was a retrospective, observational study of SNF residents between January 1, 2017–January 1,2018. The intervention group comprised residents of six urban SNF facilities in the Northeastern United States, who underwent an acute telemedicine evaluation.^11^ The telemedicine service consists of an on-demand consultation by an EP, facilitated by a clinical care specialist (CCS) who is a paramedic or emergency medical technician on-site at all times. The service is used for acute evaluations when facility staff judged that patients would otherwise require ED transfer. The CCS uses a cart with point-of-care labs, electrocardiograms, telemetry, and ultrasound (Figure 1). Patients can also be directly transported for outpatient imaging (eg, chest radiograph and computed tomography. Order sets and pathways are used to streamline decisions to treat in place or transfer. The CCS monitors SNF residents in accordance with EP orders and can re-initiate consultations. If the patient cannot be definitively managed on-site, or if the patient or family prefers transfer, the EP directs staff to carry out immediate treatments and expedite transport.

The control group consisted of residents of SNFs that did not offer telemedicine evaluations. These residents were transferred via ambulance to the ED of an urban tertiary care hospital with 55,000 visits annually. Patients were broadly matched on age and gender. The study was approved by the institutional review board of the tertiary care hospital.

### Protocol

We used electronic health record (EHR) data from the telemedicine service and the tertiary care hospital to abstract age, gender, chief complaint, and disposition. Data were de-identified in accordance with the Health Insurance Portability and Accountability Act-Safe Harbor criteria.

### Analysis

Our primary outcome was whether a patient was ultimately admitted to the hospital. For the intervention group, EHR data beyond the telemedicine visit was not available; hence, we could not definitively determine whether the patient was admitted after ED transfer. To address this limitation, we conservatively designated any patient in the intervention group who was transferred to the ED as admitted. This should underestimate the potential benefit of the intervention, as in the general Medicare population only about 30% of those treated in the ED are admitted as inpatients.^12^ The use of a full calendar-year period was intended to avoid the potential confounding effects of seasonality. The two populations were tested for demographic concordance in terms of age using an independent t-test and gender using a Fisher’s exact test, and a logistic regression was conducted with both features relative to the outcome to examine whether they played a role as confounders.

Patients in the control group were evaluated in the ED and designated as either admitted or discharged. Patients were considered discharged from the ED if they did not have an inpatient admission, or if they were directly discharged to their original facility, discharged to acute rehab, or discharged after observation care in the ED. For our primary outcome, we report the odds ratio (OR) of admission with 95% confidence interval (CI). As a significant potential benefit of telemedical care for SNFs is early intervention in chronic disease exacerbations, we conducted a subanalysis examining the OR of admission across the three most common chronic disease-related presentations found among the study population, with strict Bonferroni correction for multiple comparisons.

## RESULTS

A total of 2311 patients were evaluated in the SNF-based group, matched with 2295 patients in the control group. The groups had similar distributions by gender (intervention group: 60.2% female; control group 58.1% female; p = 0.14), but the control group was slightly older (intervention group: 75.6 [standard deviation (SD) 12.3]; control group 78.9 [SD 8.14]; p<0.001). A logistic regression demonstrated no significant association between these factors and admission. The most common reasons for telemedicine activation were exacerbations of CHF, chronic obstructive pulmonary disease (COPD), and DM (Table 1). The mean cost of the telemedicine care delivery in this study was $816 per episode.

Patients who received SNF-based acute care were less likely to have their care escalated. Only 27% of the SNF-based group were transferred to the ED, whereas 71% of the control group were admitted to the hospital from the ED (OR = 0.15 (95% CI, 0.13–0.17), p < 0.001, Table 1). These results were directionally consistent across the top three conditions, although rates of presentation for all three were significantly higher in the SNF-based group (Table 1).

## DISCUSSION

Telemedicine has been heralded as a panacea to many systemic problems in healthcare; although widespread adoption continues^13^ its proven benefits are more modest. Many studies examining telemedicine across settings have failed to find compelling clinical or cost benefits,^14^–^16^ although patients are often satisfied with these services and remain optimistic about their potential.^17^,^18^ The most successful applications of telemedicine have been subspecialty consultations in resource-limited settings. In the ED, this includes tele-neurology for acute stroke, remote radiology,^19^–^23^ and psychiatric evaluations.^24^,^25^ Telemedicine has also shown promise within SNFs for chronic disease management and related hospitalizations.

A pilot study by Dy et al demonstrated that a telemedicine team of an endocrinologist, nurse, and dietician improved glycemic control for SNF residents.^26^ Grabowski et al demonstrated a trend toward reducing unnecessary transfers by replacing SNFs’ on-call physicians with telemedicine, but had limited utilization of their service.^27^ More recently, Gillespie et al showed telemedicine reduced ED utilization for patients with dementia in senior living communities.^28^

The intervention evaluated in our study lies at the intersection of these trends, providing an EP as a specialty consultant. The potential to decrease ED transfer and hospital admission is facilitated by the CCS and expanded diagnostic tools, allowing the EP to conduct much of an ED workup in situ. Furthermore, the ability of the CCS to fulfill medication orders and re-initiate consultation effectively allows for observation care at the SNF.

While rigorous cost-effectiveness studies of telemedicine are lacking,^15^,^16^,^29^ the complexity of the interventions in this study invariably comes at increased cost. The average cost of the telemedicine service in this study was $816 per episode, compared to the flat rate of $30,000 per facility per year charged by Grabowski et al. Amortized across 2311 consultations in six SNFs over a one-year period, this represents a more than tenfold increase. Conversely, the average Medicare payment for a SNF-based rehospitalization is over $10,000.^30^ Considering the added expenses of ambulance transportation and EP fees, this enhanced telemedicine service would be cost-effective if it averted 10% of hospitalizations. The data from this program suggests an 80% reduction in care escalation, suggesting this is a worthwhile investment, irrespective of the clinical benefits from avoiding unnecessary admissions.

## LIMITATIONS

This study has several significant limitations. It is possible that the telemedicine program was activated for conditions where the staff would not automatically initiate transport to the ED, and SNFs may have substantial differences in their threshold for transferring patients; however, a similar reduction was seen in patients with COPD and CHF exacerbations, conditions where ED transfer is typically required. The lack of follow-up information for the intervention group obscures patients’ disposition after ED transfer, which we addressed by conservatively assuming these patients were admitted when many may have been observed or discharged directly. Seasonality is also a potential confounding factor, as during flu season facilities without the capacity to test or cohort patients may be more inclined to transfer patients. Finally, as a pilot study our analysis does not include specific markers of disease severity, such as oxygen saturation during COPD and CHF exacerbations, which could substantially affect the effects of the intervention. More robust matching of the groups (eg, propensity-score matching on age and comorbid conditions) would improve the generalizability of our results.

## CONCLUSION

In this pilot study, emergency physician-staffed telemedicine acute evaluations of SNF residents were associated with lower rates of hospital admissions than typical ED care, including in exacerbations of chronic diseases such as COPD and CHF, which represented a substantial portion of overall evaluations in the intervention group. The COVID-19 pandemic has broadly increased the tempo and urgency of telemedicine use; however, more in-depth studies are needed to determine whether these interventions result in longer-term reductions in chronic disease exacerbations and hospitalization rates among SNF residents. While comprehensive cost data for admitted patients was not available in this study, the reduced likelihood of hospital transport and admission for SNF residents may justify the increased upfront costs of a comprehensive telemedicine evaluation.

## Figures and Tables

**Table 1:** Care escalation processes for different conditions in telemedicine and control group.

Medical complaint and care escalation	Telemedicine group	Control group	OR (95% CI)	P-value
All conditions, n	2,311	2,295		
Care escalation, n (%)	623 (27)*	1,629 (71)†	OR 0.15 (0.13–0.17)§	< 0.001
CHF, n (% all visits)	576 (25)	314 (14)		
Care escalation, n (%)	156 (26)*	257 (82)†	OR 0.08 (0.06–0.11)§	< 0.001
COPD, n (% all visits)	607 (26)	363 (16)		
Care escalation, n (%)	158 (26)*	265 (73)†	OR 0.13 (0.10–0.18)§	< 0.001
DM, n (% all visits)	761 (33)	234 (10)		
Care escalation, n (%)	213 (28)*	152 (65)†	OR 0.21 (0.15–0.29)§	< 0.001

* Denotes transfer to the emergency department (ED)

† Denotes admission to the hospital.

§ For the purposes of this analysis it was assumed all telemedicine patients transferred to the ED were admitted; lower odds ratio indicating lower odds of admission in the telemedicine group.

*OR*, odds ratio; *CI*, confidence interval; *CHF*, congestive heart failure; *COPD*, chronic obstructive pulmonary disease; *DM*, diabetes mellitus.
